# Isolation of a nanomolar scFv inhibiting the endopeptidase activity of botulinum toxin A, by single-round panning of an immune phage-displayed library of macaque origin

**DOI:** 10.1186/1472-6750-11-113

**Published:** 2011-11-23

**Authors:** Siham Chahboun, Michael Hust, Yvonne Liu, Thibaut Pelat, Sebastian Miethe, Saskia Helmsing, Russell GA Jones, Dorothea Sesardic, Philippe Thullier

**Affiliations:** 1 Unité de biotechnologie des anticorps, et des toxines, Département de microbiologie, Institut de Recherche Biomédicale des Armées (IRBA-CRSSA), 24 avenue des Maquis du Grésivaudan, B.P. 87, 38702 La Tronche Cedex, France; 2 Institut für Biochemie und Biotechnologie, Technische Universität Braunschweig, Spielmannstr. 7, 38106 Braunschweig, Germany; 3 Division of Bacteriology, National Institute for Biological Standards and Control (NIBSC), Health Protection Agency (HPA), South Mimms, Potters Bar, Hertfordshire EN6 3QG, UK

## Abstract

**Background:**

Botulinum neurotoxin A (BoNT/A), mainly represented by subtype A1, is the most toxic substance known. It causes naturally-occurring food poisoning, and is among the biological agents at the highest risk of being weaponized. Several antibodies neutralizing BoNT/A by targeting its heavy chain (BoNT/A-H) have been isolated in the past. For the first time however, an IgG (4LCA) recently isolated by hybridoma technology and targeting the BoNT/A light chain (BoNT/A-L), was shown to inhibit BoNT/A endopeptidase activity and protect in vivo against BoNT/A. In the present study, a phage-displayed library was constructed from a macaque (*Macaca fascicularis*) hyper-immunized with BoNTA/L in order to isolate scFvs inhibiting BoNT/A endopeptidase activity for clinical use.

**Results:**

Diversity of the scFvs constituting the library was limited due to the frequent presence, within the genes intended to be part of the library, of restriction sites utilized for its construction. After screening with several rounds of increasing stringency, as is usual with phage technology, the library got overwhelmed by phagemids encoding incomplete scFvs. The screening was successfully re-performed with a single round of high stringency. In particular, one of the isolated scFvs, 2H8, bound BoNT/A1 with a 3.3 nM affinity and effectively inhibited BoNT/A1 endopeptidase activity. The sequence encoding 2H8 was 88% identical to human germline genes and its average G-score was -0.72, quantifying the high human-like quality of 2H8.

**Conclusions:**

The presence of restrictions sites within many of the sequences that were to be part of the library did not prevent the isolation of an scFv, 2H8, by an adapted panning strategy. ScFv 2H8 inhibited toxin endopeptidase activity in vitro and possessed human-like quality required for clinical development. More generally, the construction and screening of phage-displayed libraries built from hyper-immunized non-human primates is an efficient solution to isolate antibody fragments with therapeutic potential.

## Background

*Clostridium botulinum*, and certain other *Clostridium spp.*, secrete seven serotypes (A-G) of botulinum neurotoxins (BoNTs). Three BoNTs (A, B and E) are mainly responsible for human botulism, a disease occurring naturally, in the form of food poisoning. Botulism is also part of the A list of the six diseases at the highest risk of being caused by bioweapons, according to the Center for Disease Control [1]. Botulinum toxin A (BoNT/A) is regarded as the most toxic substance on Earth and its LD_50 _values are 1 ng/kg for the intravenous and subcutaneous routes, and 3 ng/kg by pulmonary route [2]. Botulinum toxins exert their toxicity by cleaving proteins that constitute the intraneuronal SNARE (soluble N-ethylmaleimide-sensitive factor attachment protein receptor) complex, which allows cholinergic vesicles to bind the pre-synaptic membrane of neuromuscular synapses and release their content. In particular, BoNT/A cleaves SNAP-25 (synaptosomal-associated protein 25 kDa), due to a zinc metalloprotease activity borne by its light chain (BoNT/A-L). This proteolysis inhibits SNARE activity and causes flaccid paralysis, including that of respiratory muscles. Despite botulinum lethality, vaccination against botulinum toxins is questionable because it would prevent the ever-increasing medical uses of BoNTs (for a review of these uses, see [3]; for a discussion on the limits of vaccination against botulinum toxins see [4]). At present, treatment against botulism consists of supportive care and passive immunization with equine antitoxin [5], which may however cause hypersensitivity and serum sickness [6]. To avoid these side effects and increase their half-life, particularly for prophylactic use, well-tolerated antibodies are needed. These may be represented by recombinant antibodies.

Antibodies neutralizing botulinum toxins generally target the heavy chains of these toxins, inhibiting toxin entry into cells [7-14]. Recently however and for the first time, an antibody directed against the light chain of botulinum A (BoNT/A-L), the human IgG 4LCA isolated by hybridoma technology, was shown to neutralize the proteolytic activity of BoNT/A in vitro and exhibited protective activity in vivo. Moreover, when 4LCA was administered in conjunction with an antibody directed against the heavy chain, both acted synergistically and showed increased protective capacities [15]. A llama antibody also inhibiting BoNT/A-Lc enzymatic activity was presented even more recently, and its epitope was mapped to support the design of synthetic inhibitors [16]. In the present study, we describe the isolation of a human-like recombinant scFv inhibiting BoNT/A endopeptidase activity *in vitro*, in the perspective of its clinical development.

In previous studies, we have used immune phage-displayed libraries originating from macaques (*Macaca fascicularis*) to isolate antibody fragments of nanomolar or picomolar affinities against tetanus toxin [17], the two units of anthrax lethal toxin [18,19], ricin [20], and against a surface antigen of *Aspergillus fumigatus *[21]. The choice of non-human primates (NHPs) is based on the phylogenetic proximity between NHPs and humans. This choice allows the isolation of fragments with human-like character, thus augmenting their therapeutic value. At a later stage, the best NHP antibody fragments might be germline-humanized to obtain antibody fragments with a higher percentage of identity with human germline sequences than antibody fragments of human origin, thus potentially better tolerated [22-24]. Another potential advantage of our strategy is that we choose animals from which, prior to the immunization, no DNA encoding antibody fragments are amplified so that the library is not directed against non-relevant antigens, but rather exquisitely focused on the immunogen. From this "zero" point, the immunization is conducted until a high titer is reached and can not be increased with additional immunizations. Such a response is defined as hyper-immune [25] and may span over most, or all, epitopes with high-affinity antibodies. Because hyper-immune NHPs are used for the construction of our libraries, we have argued that they could be referred to as "hyper-immune libraries" [26]. In the present study, the strategy of utilizing a hyper-immune NHP library was chosen again. Diversity of the scFvs constituting this library was however limited by the frequent presence of NcoI and Hind III restriction sites within the genes encoding scFvs and utilized for library construction. After multi-step panning, phages displaying incomplete scFvs overwhelmed the library. A single-round of panning with high stringency was more successful and several full-sized scFvs were isolated, whose human-like character was quantified. One of these scFvs inhibited the endopeptidase activity of BoNT/A and may be proposed for clinical development. This study exemplifies again the potential of our strategy, and indicates how to solve difficulties similar to the problem encountered here.

## Results

### Animal immunization, phage-displayed library construction

The immunization utilized the non-toxic light chain of botulinum A1 (BoNT/A1-L) as immunogen, but the response was evaluated with an ELISA utilizing the whole toxin (BoNT/A1) as capture antigen because BoNT/A1 better represents the agent to be neutralized in clinical use. Ten days after the third injection, the ELISA titer was measured as 1/100 000 and was anticipated to correspond to an hyper-immune response. A fourth injection was given to the macaque eight months after the third one and, just before this, fourth injection, no PCR products could be amplified from the bone marrow. At the sixth and tenth days after this fourth injection however, the amplification of genes coding VH and VL was very effective with all primers, and then the quantity of the amplified products decreased. On the tenth day, the titer was measured again as 1/100 000, confirming that a stable, hyper-immune, response had been reached. The genes encoding VH and VL which had been amplified during the sixth and tenth days were regarded as optimal. They were pooled and cloned in pGemT for the construction of κ light chains and Fd sublibraries of 10^4 ^and 5 × 10^4 ^clones, respectively. Next, the scFv library was constructed in pHAL14 by two consecutive cloning steps, starting with the VL gene fragments and followed by the VH fragments. The final scFv antibody gene library consisted of 3.3 × 10^8 ^independent clones, but with only 37% full size inserts as determined by colony PCR. Twenty clones of the library were sequenced prior to screening, and the BLAST analysis (http://www.ncbi.nlm.nih.gov/blast/Blast.cgi) of these sequences revealed that only seven of them corresponded to full-sized scFvs. Partial sequences corresponded to internal *Nco*I restriction sites in 8 of 20 VH gene fragments, plus a *Hind*III restriction site on one. The existence of partial VH gene fragments, possibly corresponding to recombination in 4 occurrences (data not shown), was also observed. Despite this low rate of full-sized inserts (7/20 or 35%), the immune library was packaged.

### Isolation of scFvs directed against BoNT/A1

A single round of panning followed by forty washes, was performed and resulted in the isolation of 2.4 × 10^4 ^clones. Randomly chosen, 288 individual clones were grown in SB medium, and culture supernatants were tested for binding to BoNT/A1 by ELISA. Thirty nine positive clones expressing scFvs reacting with BoNT/A1 were isolated and the corresponding DNA was sequenced. These 39 clones corresponded to 14 non-redundant, full-sized scFvs.

### Affinity measurement

From the 14 clones selected previously, 10 scFvs were efficiently expressed and purified before measuring their affinities for BoNT/A. These scFvs had nanomolar affinities, from 1.52 nM (2A3) to 7.57 nM (3C6) (table 1), with an average value of 3.9 nM.

**Table 1:** Affinities of the 10 selected scFvs.

scFv	K_D _(nM)
2A3	1.52
1G7	7.5
2A6	5.26
2F2	1.64
2G10	3.32
2H8	3.3
3C6	7.57
3C11	2.8
3G12	4.13
2D1	1.65

Affinities of the ten scFvs reacting with BoNT/A1 as measured by Biacore.

### Computational analysis

Analysis of the genes encoding the 10 scFvs indicated a high diversity of the VH sequences, despite the loss of diversity due to panning and presence of the *Nco*I and *Hind*III sites. All IGHV genes were represented, except for IGHV2, combined with representatives of all IGHD genes except for IGHD4 (Table 2). These genes were only combined with representatives of IGHJ4 and IGHJ5 but, as these genes only encode the last part of CDR3 and FR4, this limited diversity of the J genes was not expected to severely impact diversity of the VH paratopes. Diversity of the VL part of the paratope was however smaller with only IGKV1 being represented but for one occurrence of IGKV3 and one occurrence of IGKV4. IGKJ1 was dominant, again limiting VL diversity, except for two occurrences of IGKJ3 and two occurrences of IGKJ4. The germinality index (GI), indicating percent identity between framework regions (FRs) of the selected scFvs and those coded by their most similar human germline genes, was calculated. It was averaged on VH and VL for each scFv and ranged from 82% to 88% (Table 2). The G-scores, another indirect way to predict tolerance but based on a comparison with somatic (expressed) genes, ranged from -2.689 to 0.518 (Table 3).

**Table 2:** Human germline genes most similar to the 10 selected scFvs.

	VH			VL		
	
scFv	V	D	J	V	J	GI (%)
2A3	IGHV4-28*01	IGHD3-22*01	IGHJ4*02	IGKV1D-13*01	IGKJ3*01	88

1G7	IGHV3-11*03	IGHD2-2*03	IGHJ5*02	IGKV1-6*01	IGKJ4*01	82

2A6	IGHV4-4*03	IGHD5-12*01	IGHJ4*02	IGKV1-9*01	IGKJ1*01	88

2F2	IGHV1-8*01	IGHD6-25*01	IGHJ5*02	IGKV1-39*01	IGKJ1*01	87

2G10	IGHV3-66*02	IGHD3-9*01	IGHJ4*02	IGKV1-33*01	IGKJ1*01	85

2H8	IGHV1-2*02	IGHD2-8*02	IGHJ5*02	IGKV3-11*01	IGKJ2*04	88

3C6	IGHV5-51*01	IGHD1-1*01	IGHJ5*02	IGKV1-12*01	IGKJ4*01	84

3C11	IGHV1-2*02	IGHD2-8*02	IGHJ5*02	IGKV1-5*03	IGKJ1*01	86

3G12	IGHV3-23*04	IGHD6-13*01	IGHJ4*02	IGKV1-27*01	IGKJ3*01	86

2D1	IGHV4-61*02	IGHD6-13*01	IGHJ4*02	IGKV4-1*01	IGKJ4*01	85

Human germline genes most similar to the genes coding for the 10 selected scFvs were retrieved by IMGT /DomainGapAlign. The germinality index (GI) of each scFv (VH+VL) is indicated in the right column.

**Table 3:** G-scores of the 10 selected scFvs.

scFv	chain	G-score
2A3	VH	-0.826
	
	VL	-0.937

1G7	VH	-0.562
	
	VL	-2.689

2A6	VH	-1.201
	
	VL	-0.879

2F2	VH	0.518
	
	VL	-1.011

2G10	VH	-0.709
	
	VL	-0.812

2H8	VH	-0.530
	
	VL	-0.901

3C6	VH	-2.124
	
	VL	0.070

3C11	VH	-0.310
	
	VL	-0.541

3G12	VH	-1.199
	
	VL	-0.898

2D1	VH	-1.341
	
	VL	-0.845

The human-like character of the 10 selected scFvs was evaluated through the calculation of G-scores, to indirectly predict their immunogenicity.

### Enzymatic assays

**Figure 1:**
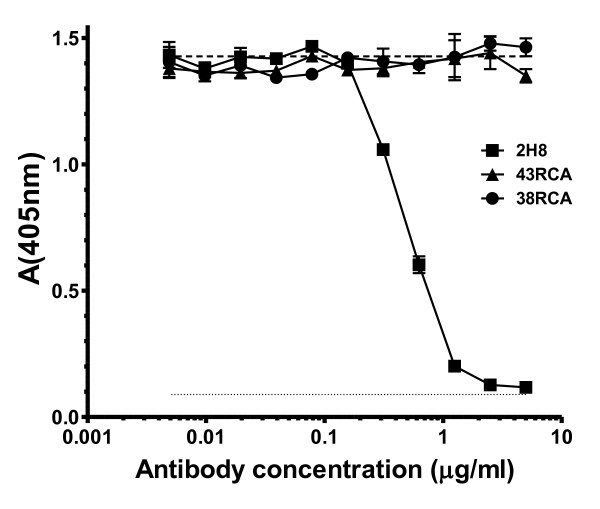
**Neutralisation of 10LD_50_/ml BoNT/A with scFv 2H8**. 2H8 reacted with BoNT/A1 (43.5 pg/ml; 10 LD50/ml) and showed dose dependent inhibition of endopeptidase activity in vitro. The negative controls (43RCA and 38RCA), directed against ricin toxin, showed no inhibition at equivalent protein concentration. Results are mean with range (n = 2). BoNT A1 toxin control (10 LD50/ml): broken line. Buffer control: dotted line.

In a preliminary assay, 2H8 was the only scFv to present a distinct level of endopeptidase inhibition while all other clones showed almost no inhibition of proteolysis, comparable to that of the negative controls, scFvs 43RCA and 38RCA directed against ricin. Consequently, 2H8 was more precisely evaluated for its inhibition capacity (Figure 1) in a second experiment, in which a concentration of 2H8 equal to 0.5 μg/ml (18.5 nanomolar) inhibited 50% of the endopeptidase activity of 43.5 pg/ml of BoNT/A1 (0.29 picomolar). Inhibition of 50% of the endopeptidase activity thus corresponded to a molar ratio of antigen binding sites/antigen (2H8:BoNT/A1) of 64,000:1 (0.0185 10^-6^:0.29 10^-12^).

## Discussion

Botulinum toxins (BoNT) are the most poisonous substances on Earth, and botulinum neurotoxin A (BoNT/A) is the most potent. In a broad study, sixty six genes encoding BoNT/A were studied [27] and subtype 1 was encountered on 54 occurrences, with the remaining strains scattered among 3 other subtypes. This study thus focused on the most prevalent BoNT/A subtype, A1. We targeted its light chain (BoNT/A1-L) because several antibodies targeting the heavy chain, and inhibiting toxin entry into cells, have already been isolated [7-14]. The description of BoNT/A neutralization by targeting BoNT/A-L and inhibiting its endopeptidase activity is fairly recent, and to date only one human (4LCA) and one llama (Aa1) antibody with this capacity have been isolated [15,16]. In confocal microscopy, signals corresponding to 4LCA co-localized in cells with signals corresponding to BoNT/A, representing direct evidence that 4LCA did not act by preventing BoNT/A internalization and that 4LCA was internalized for neutralization [15]. Also, the structure of Aa1 complexed with BoNT/A-L, and the epitope of Aa1 on BoNT/A-L, was determined by crystallography [16]. Inhibition of endopeptidase activity by antibodies directed against the light chain of BoNT/A is thus thought to mainly depend on their direct interaction, initiated outside of the cells but persisting within. Such a mechanism of inhibition has the advantage that it may be activated after entry of the toxin in the cells so that antibodies with this activity might be proposed for re-formatting as transbodies or intrabodies [28], which would enter cells even if not complexed with the toxin and thus would be usable after a longer delay than antibodies that prevent toxin cell entry. In addition, one may anticipate that an antibody inhibiting endopeptidase activity may also have other protective mechanisms in vivo, such as promoting toxin clearance by recruitment of immune effectors [29]. For these reasons, it was decided to isolate an antibody fragment inhibiting BoNT/A1-L endopeptidase activity, in anticipation of its protective capacity and potential clinical development.

The present library was constructed as previously described [18,19,21] to yield high affinity-scFvs [17] due to its hyper-immune nature [30], corresponding to the hyper-immune response from which this library was built. Non-toxic BoNT/A1-L was utilized for immunization but complete BoNT/A1 was preferred as antigen for ELISAs, panning, affinity measurements and endopeptidase assay as the present study aimed at isolating scFvs that bind to BoNT/A1-L but as part of BoNT/A1, the toxin to be neutralized. After library construction however, internal controls showed an unusually low rate of inserts (37%), which was confirmed by sequencing as almost half (8/20) of inserts presented an internal *Nco*I restriction site. An *Hind*III internal restriction site was also observed, plus partial sequences probably caused by recombination, so that only 35% (7/20) of scFv sequences were full-length. Former macaque immune libraries [19-21] did not exhibit this rate of partial VH gene fragments as apparently, a higher rate of VH gene fragments with *Nco*I restriction sites was employed by the immune response against BoNT/A1-L compared to all former immunizations. Notwithstanding, the constructed library was screened using a traditional protocol consisting of successive rounds of panning, with increasing stringency due to an increasing number of washing steps between rounds. After the first round, with 5 washing steps, the presence of full-sized inserts further decreased. This decrease was due to the library being overwhelmed by plasmids carrying partial-sized inserts or no insert at all, which replicate more rapidly and this preferred replication had not been counterbalanced by the selection applied during the first round of panning. After the second round, with 10 washing steps, scFvs with partially- and full-sized inserts were completely lost and the panning was thus unsuccessful. However, it was anticipated that NHP hyper-immune libraries do not absolutely need multi-step panning, in contrast to naïve libraries where scFvs of interest are rare, so that it was decided to adapt the panning strategy and use only a single round of panning, similar to other studies [31-33]. A high stringency, in the form of 40 washes, was chosen for this single round because it was required to counterbalance the preferred replication of phages with incomplete, or no, scFvs. Also, it was expected that scFvs of interest, present in the library, would have a high affinity and thus would be retained even after 40 washing steps. This single-round panning allowed the elution of clones, of which two hundred and eighty eight were randomly hand-picked and small-scale expression of scFvs was induced. The supernatants were tested in ELISA for their reactivity against BoNT/A1 and thirty nine scFvs were rated as positive. The numerous negative supernatants could correspond to plasmids carrying no or partial inserts and again beginning to overwhelm the library, to scFvs with weak affinities or to scFvs having a low expression level in this format. After sequencing these thirty nine positive clones, fourteen full-sized, non redundant scFvs were isolated. Of these fourteen, ten were expressed and purified in quantities sufficient for further evaluation. The sequencing of these ten scFvs showed a high level of diversity, most particularly in the VH region. Given that diversity, it was expected that these ten scFvs might target different epitopes and perhaps inhibit endopeptidase activity. This was the case for only one of them, 2H8, which bound the BoNT/A1 holotoxin with high affinity (3.3 nM) and inhibited SNAP25 cleavage. The inhibition capacity of 2H8 might be compared to that of the only two antibodies with similar activities, previously published. The molar ratio of antibody to toxin (antibody:toxin), at which 50% inhibition of endopeptidase activity is observed, is the best parameter to evaluate an inhibiting activity as this ratio is independent of toxin and antibody quantities utilized in the test. In a previous study, the IgG 4LCA was tested in an in vitro endopeptidase assay but at a single dose, allowing 63% reduction of BoNT/A endopeptidase activity [15]. This dose corresponded to a 10,000:1 molar ratio (antigen binding sites of IgG 4LCA:BoNT/A), which is comparable to the 64,000:1 molar ratio observed when 2H8 inhibited 50% of the BoNT/A endopeptidase activity. In effect, scFv 2H8 has a smaller steric hindrance than an IgG thus the expression of 2H8 as an IgG should lower that ratio. In another study, the llama antibody Aa1 inhibited 50% of BoNT/A-L endopeptidase activity at a 200:1 molar ratio (Aa1:BoNT/A-L) [16]. However, it should be noted that Aa1 was tested only against the BoNT/A light chain (BoNT/A-L) and not the whole toxin. In the view of clinical development, such a test is less realistic than an evaluation utilizing BoNT/A, as was performed with 4LCA and with 2H8.

The G-score of a variable domain (VH or VL) characterizes the human-like character, or humanness, of this domain. Assuming a high level of humaness corresponds to a high degree of tolerance in clinical use, as is generally accepted [34-36], the G-score parameter indirectly predicts tolerance of the variable domains it characterizes. Here, the G-score of 2H8 VL (-0.901) corresponds to a VL that is "as human" (as similar to human VLs) as 19% of the human VLs present in the Kabat database. Similarly, its VH, with a G-score equal to -0.530, is "as human" (as similar to human VHs) as 29% of human VHs. For both V-regions, these similarities testify for the human-like character of 2H8 and predict good tolerance in clinical use. Another way to evaluate the immunogenicity of 2H8 is to calculate the identity level of its FRs with its most similar, human, germline-encoded FRs. This identity level, also called germinality index (GI) [18], indirectly predicts the tolerance of any given VH or VL sequence based on the theoretical assumption that germline-encoded sequences are best tolerated, as they are part of the "immunological self". The GI value of 2H8 is equal to 88%, thus high. For instance, this value is identical to the GI value of another macaque antibody fragment, 35PA_83 _[18], which was germline-humanized up to a GI of 97.8% [22]. This last GI value corresponded to only four macaque residues that had to be left after the humanization process, with the rest of the FRs encoded by germline genes. Only two of these four residues were exposed to solvent, and thus unlikely to raise any immune response in humans. Based on the GI identity of 2H8 with 35PA_83_, success of germline-humanization of 2H8 may be anticipated. Overall, the sequence analysis of 2H8 by G-score and GI quantifies the human-like character of 2H8, and predicts its good tolerance. To suppress any risk of immunogenicity however, germline-humanization of 2H8 is recommended as it should be as successful as a germline-humanization, presented formerly [22].

## Conclusion

In the present study, scFv 2H8, directed against the light chain of botulinum toxin A, was isolated. It presented an inhibition capacity and human-like qualities required for clinical development. ScFv 2H8 was isolated despite the presence of restriction sites utilized for library construction within the genes encoding the scFv intended to be part of the library, using an adapted single-round panning strategy. This result exemplifies the efficiency of phage-displayed libraries built from immunized non-human primate to isolate antibody fragments with therapeutic potential.

## Methods

### Ethical Statement

The animal experiment was approved and performed in compliance with all relevant French ethical guidelines and laws, in particular (i) « partie règlementaire du livre II du code rural (Titre I, chapitre IV, section 5, sous section 3: expérimentation sur l'animal) », (ii) « décret 87-848 du 19-10/1987 relatif aux expériences pratiquées sur les animaux vertébrés modifié par le décret 2001/464 du 29/05/2001 », (iii) « arrêté du 29 octobre 1990 relatif aux conditions de l'expérimentation animale pour le ministère de la défense » and (iv) « instruction 844/DEF/DCSSA/AST/VET du 9 avril 1991 relative aux conditions de réalisation de l'expérimentation animale ».

### Animal immunization

Four subcutaneous injections of the botulinum neurotoxin light chain A1 (BoNT/A1-L) (Metabiologics Inc., Madison, Wi, USA) were administered with a one month-interval, except for the fourth injection given with an eight-month interval, to a male cynomolgus macaque (*Macaca fascicularis*). The first injection consisted of 80 μg of the light chain of BoNT/A mixed with complete Freund's adjuvant (Sigma, Isle d'Abeau, France) and for the remaining injections, incomplete Freund's adjuvant was used.

### Construction of the anti-BoNT/A1-L antibody library

RNA was isolated from the bone marrow using Tri Reagent (Molecular Research Center Inc, Cincinnati, USA), and was retro-amplified. Seven primers were used to amplify DNA encoding κ light chains, and nine primers were used to amplify of DNA encoding Fd fragments of the γ chain [30]. To obtain two sub-libraries encoding these light chains or Fd fragment, the corresponding PCR products were pooled and subcloned in the pGemT vector (Promega, Madison, Wisconsin). Later, these PCR products were reamplified as DNA encoding the VL (variable region of the light chain) or VH (variable region of the heavy chain) regions only, with two oligonucleotide primer sets introducing restriction sites as described elsewhere [30]. During the first step of the library construction, the VL fragments were inserted into pHAL14 [21,37,38], and then the VH fragments were inserted into pHAL14 containing the VL repertoire. For this, the pHAL14 vector and the VL fragments were digested with MluI and NotI (New England Biolabs, Frankfurt, Germany), the enzymes were inactivated, pHAL14 was dephosphorylated using calf intestinal phosphatase (MBI Fermentas), and the DNA was purified. VL PCR products (270 ng) were inserted into 1 μg of the dephosporylated pHAL14 preparation in four separate ligation reactions. DNA was precipitated from the reaction mixes with ethanol and sodium acetate, the pellet was washed twice with 70% ethanol, and then four aliquots (25 μl) of XL1-Blue MRF' (Stratagene, Amsterdam, The Netherlands) were used for electroporation. Plasmids (the VL library) were isolated using a Plasmid Midi Kit (QIAGEN, Hilden, Germany). The VL library and the VH fragments were digested with NcoI and HindIII (New England Biolabs), and ligation and electroporation were then performed as described for VL. The library was packaged using hyperphages [39,40].

### Screening of the library against BoNT/A1

A plate (Nunc, Roskilde, Danemark) was coated with holotoxin BoNT/A1 (Metabiologics, Madison, Wisconsin) at a concentration of 20 μg/ml in PBS (ON, 4°C). The plate was then saturated with TBS/BSA 3% (2 hours, 37°C) and phages were incubated (2 hours, 37°C) in the wells. One single round of panning composed of forty washes was then performed using TBS/Tween 0.1% as the washing buffer, with an interval of 5 min between each wash [41]. The plate was finally rinsed with sterile TBS and the phages were eluted with trypsin (10 mg/ml in TBS, incubated at 37°C, 30 min). The eluted phages were used for infecting *E. coli *(SURE strain, Stratagene) previously cultured in SB (Super Broth) medium supplemented with tetracycline (10 μg/ml). Infected *E. coli *were later grown in Petri dishes (ON, 37°C).

### ScFv production and purification, ELISA and affinity measurements

ScFvs were expressed following a previously described method [19] but purified on a His Trap column (GE Healthcare, Buckinghamshire, UK) using a Profinia automate (Biorad, Hercules, California), according to the manufacturer's instructions. Purity was verified and scFvs were quantified using capillary electrophoresis on an Experion device (Biorad). For ELISA, plates were coated as for panning and the rest of the procedure was performed as previously described [19]. Affinities were measured by surface plasmon resonance with a BIAcore X (General Electric-Biacore, Uppsala, Sweden) instrument. Holotoxin BoNT/A1 was immobilized at a maximum of 800 resonance units on a CM5 chip (General Electric-Biacore) via amine coupling, according to the manufacturer's instructions. A flow rate of 30 μl/min was maintained during measurements. For each measurement, a minimum of six scFv dilutions (10 to 0.1 μg/ml) in HBS-EP buffer (General Electric-Biacore) were each tested for 1000 s. After each scFv dilution, the chip was regenerated with glycine 1.5 (General Electric-Biacore), run at 10 μl/min for 30 s. Constants were calculated using the Biaevaluation software (Biacore) [42] and were verified by internal consistency tests [43].

### DNA sequencing, sequence analysis

Twenty scFvs were isolated before panning and sequenced by GATC Biotech (Konstanz, Germany). Thirty nine scFvs were isolated after panning and sequenced by Beckman Coulter Genomics (Takeley, United Kingdom). Sequences of the scFvs isolated after panning were compared with human germline sequences, using the IMGT/V-QUEST tool, available on-line from the International ImmunoGeneTics information system^® ^(IMGT) (http://www.imgt.org.) [22]. This tool finds the human germline sequences most similar to any given variable region and also calculates its Germinality Index (GI), defined as the percent identity between its FRs and those encoded by its most similar human germline sequences. Human germline genes encode proteins belonging to the "immunological self", thus the GI evaluates the degree of identity of any given variable region with the human "immunological self" to indirectly quantify its immunogenicity [22]. For the time being however, there is no benchmark regarding GI of human antibodies. This is in contrast with G-score, which evaluates the percentage of identity of any given VH or VL with all human expressed VH or VL, respectively, belonging to the same family and present in the latest release of the Kabat database. G-score is also available on line (http://www.bioinf.org.uk) to predict potential immunogenicity, indirectly but by comparison with somatic, not germline, sequences. It is an evolution from the former H-score (originally called Z-score) [24,44]. The G-score is zero for a VH or VL sequence having the same identity level with its human counterparts than the average identity level of human VH or VL sequences present in Kabat's database, respectively. Positive scores correspond to higher than average identity level, and conversely for negative scores.

### Enzymatic assay

Previously published in vitro endopeptidase immuno-assay for BoNT/A toxin [45] was modified and used to assess inhibitory properties of the scFvs. In this assay, the curve presenting ELISA signal as a function of BoNT/A endopeptidase activity has a linear part, utilized in the present study, where a 50% signal decrease corresponds to a 50% decrease in endopeptidase activity. Here, BoNT/A1 was purchased from Metabiologics (lot # A062805-01 with specific activity of 2.3 × 10^8 ^mouse i.p. LD_50_/mg). This toxin preparation was diluted to a stock concentration of 87 pg/ml (20 LD_50_/ml) in reaction buffer (50 mM HEPES, 20 μM ZnCl_2_, pH7.0, 0.5% (v/v) Tween 20, 5 mM DTT). All scFvs were diluted to 10 μg/ml in reaction buffer and 150 μl was added to all wells in column 1 of a 96-well low binding polypropylene plate (Nunc). 75 μl of reaction buffer was then added to all wells in columns position 2 to 11. Twofold dilutions of antibody samples were performed across the plate, initiated by removing 75 μl from wells in column 1 and completing by discarding 75 μl from wells in position 11. The stock solution of BoNT/A (75 μl) was added to all the wells to provide a final concentration of 43.5 pg/ml toxin, corresponding to 10 LD_50_/ml. The toxin/antitoxin mixture was briefly shaken on a plate shaker for 1 min at room temperature, then incubated for 1 hr at 37°C, before being transferred (50 μl/well) to another 96-well ELISA plate previously coated with custom made SNAP-25(137-206) peptide. The plate was incubated for ~18 hr at room temperature and washed 3 times in PBS-0.05% (v/v) Tween 20 (wash buffer). Specific cleavage of SNAP-25 (137-206) peptide by the BoNT/A toxin was detected with a commercial anti-SNAP-25 mouse monoclonal antibody (Lifespan Biosciences LS-C39152/10719), diluted 1:750 in wash buffer containing 2.5% (w/v) skimmed milk powder, of which 100 μl were added to all wells. The plate was incubated at room temperature for 90 mins, washed and reaction visualised by addition of 100 μl/well of diluted anti-mouse-HRP conjugate (Sigma), diluted 1:2000 in wash buffer containing 2.5% (w/v) skimmed milk powder) [45].

## Abbreviations

BoNT: botulinum neurotoxin; FR: framework regions; GI: germinality index; IgG: immunoglobulin G; NHP: non-human primate; scFv: single chain fragment variable; VH: variable domain of the heavy chain; VL: variable domain of the light chain.

## Authors' contributions

SC and TP performed the majority of bench work on the library, except for the library cloning in pHAL14. SM, SH and MH cloned the library in pHAL14, analysed its content before panning and wrote the corresponding paragraphs. YL, RGAJ and DS performed the neutralization assay and wrote the corresponding paragraphs. PT conceived the study, adapted the panning strategy and wrote the manuscript except for library construction and neutralization assay. All authors read and approved the final manuscript
